# Accidental ingestion of swamp stone: A new and hazardous source of lead poisoning

**DOI:** 10.1002/ccr3.4875

**Published:** 2021-10-26

**Authors:** Abbas Aghabikloo, Nazanin Zamani

**Affiliations:** ^1^ Department of forensic medicine and toxicology Iran University of medical sciences Tehran Iran; ^2^ Resident of Internal Medicine Department of Internal Medicine School of Medicine Hazrat‐ e Rasool General Hospital Iran University of Medical sciences(IUMS) Tehran Iran

**Keywords:** lead poisoning, swamp stone, treatment

## Abstract

It is important to find sources for lead poisoning and educate the population about the danger of this heavy metal. The epidemiologic studies for evaluating BLL can help develop a guideline for screening endangered populations and providing treatment.

## Graphical abstract

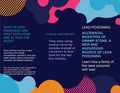

## 1 INTRODUCTION

Lead is a heavy metal that is widely used in industries. Lead poisoning is a global medical problem. The primary sources of toxicity are contaminated water and soil. Other sources are opium, fake lipstick, and color materials made from lead.

You can find "swamp stone," "Sang‐e‐Mordab," or so‐called "Silver stone" in most Iranian perfume shops. It is a powder rubbed on the skin as a deodorant to remove underarm odor and reduce sweating—for example, sweaty feet. The powder is obtained by pulverizing the silver stone, consisting of nearly 70% heavy metals, mostly lead oxide (PbO). Since lead oxide is highly absorbent through the skin and gastrointestinal tract, excessive use or ingestion of this powder can cause poisoning.

Most common sources of lead poisoning (LP) include occupational, cosmetics, food and water supply, opium consumption, old pipes, and leaded dishware.^1^ Lead can affect the central and peripheral nervous system, skin, kidneys, reproductive system, gastrointestinal system, and musculoskeletal system. Patients with high blood lead levels may experience severe, intractable abdominal colic, motor clumsiness, clouded consciousness, weakness, and paralysis.^2^

The prenatal and early life exposures to lead are associated with lower IQ, antisocial behavior, and attention‐deficit hyperactivity disorder (ADHD). Although LP is primarily asymptomatic in children, high lead levels can irreversibly impair neurocognition and behavioral development.^3^ A high blood lead level in adults is associated with decreased memory, attention, and loss of vision.^4^ Most patients experience anemia; basophilic granulation in red blood cells is one of the specific signs of LP in peripheral blood smears.^5^ Lead as a partial agonist of calcium‐binding receptors can affect neuronal signaling.^6^

Moreover, patients with chronic LP showed impaired renal function and high blood pressure.^7^ LP can reduce male sperm count and cause loss of libido and hormonal imbalance.^8^, ^9^ In females with high lead levels, the rate of stillbirths, neonatal deaths, and infertility is significantly increased.^10^

In recent years, LP has become a widespread public health concern in the United States. For example, in the Flint water crisis in Michigan, the heavy metal levels in drinking water became significantly elevated due to lead pollution from aging pipes.^11^

Over the past years, many organizations and communities have developed guidelines and protocols to manage this crisis better and help the populations in danger. The World Health Organization and the Centers for Disease Control and Prevention have declared that no blood lead level is safe.^12^ Since there is no official organization in Iran for investigating LP, the average lead level in the Iranian population is not well studied. Therefore, practitioners face many difficulties while diagnosing and treating these patients. In the current study, we present a family of five with different signs and symptoms of LP from swamp stones.

## 2 CASES PRESENTATION

A family of five (Table 1) visited the LP clinic complaining of mostly fatigue and exhaustion. They were using swamp stone by mistake instead of cinnamon in their food for the last five months. The mother was the most symptomatic, presenting with recurrent abdominal colic, loss of appetite, nausea, fatigue, joint pain, generalized muscle pain, paresthesia, and edema in the extremities. The mother claimed that she consumed more swamp stone powder than other family members as she used it instead of cinnamon in her herbal tea by mistake.

**TABLE 1:** Demographic variables

	Father	Mother	Older child	Middle child	Younger child
Gender	male	Female	Female	Female	male
Age(years)	50	45	25	17	8
Occupation	Employee	housewife	student	student	student

**TABLE 2:** Signs and symptoms on arrival

Signs and symptoms	Father	Mother	Older child	Middle child	Younger child
Paresthesia	P*	P	A**	A	A
Arthralgia	A	P	P	A	A
Muscles Pain	P	A	A	P	A
Muscles Weakness	P	P	P	P	P
Cognitive deficit	A	A	A	P	P
Memory loss	A	A	A	P	P
Abdominal pain	P	P	P	P	P
Nausea	P	P	P	P	P
Diarrhea	P	P	P	P	P
Loss of appetite	P	P	A	A	P
Anemia	A	P	A	P	A

* Present;

** Absent.

They presented with complaints of an unusual taste, loss of appetite, paresthesia, weakness, abdominal pain, and gastrointestinal discomfort. The older daughter was complaining of shortness of breath and dyspnea as the initial symptom. Parents both were experiencing severe paresthesia in the upper and lower extremities. The mother and the middle child complained of a depressed mood. Loss of appetite and weight loss were the most prominent complaints of the father. The mother and younger daughter were anemic. The mother and oldest daughter had arthralgia, especially in their wrist. Their son had some macular red skin color changes on his hand and foot. The summary of their signs and symptoms is explained in Table 2. The result of the initial blood lead level is available in table three. For initial evaluation, we took blood and urine sample from all family members and check for hematologic and metabolic factors. Table 3 presents the results of blood samples.

**TABLE 3:** Hematologic and metabolic results

	Father	Mother	Older child	Middle child	Younger child
WBC	5.3	8.9	8.6	11	9.2
Hb	14.7	8.4	12.4	10.3	11.8
MCV	85	82.7	80.2	78.9	77.3
Plt	177	253	239	195	281
Urea	45	36	17	27	22
Cr	1.1	0.7	0.7	0.7	0.6
Na	149	136	132	136	138
K	3.6	4.2	3.2	3.9	3.8
Ca	9.7	10.5	9.3	10.5	10.1
Alb	5.2	4	4.4		
AST	25	46	12	18	21
ALT	40	73	11	12	13
ALKp	91	110	101	155	542
Bili T	1.3	0.7	1.5	0.5	0.7
Bili D	0.5	0.2	0.4	0.2	0.2
U/A	Normal	Normal	Normal	Normal	Normal
T3	1.13	1.13	1.4	1.22	
T4	5.49	12.5	10.44	10.9	
TSH	10.3	2.11	2.75	2.94	
Vit D	14	32	20.3	14	
Iron	180	201	109		
Ferritin	210	123	35		
G6PD	sufficient	sufficient	sufficient	sufficient	sufficient

We admitted all of them to the hospital to receive intravenous chelation therapy. We treat four older family members with intravenous ethylenediaminetetraacetic acid (CaNa2 EDTA), two grams daily for 3 days. The younger child received only one gram daily (25 mg/kg/day) of EDTA for three days. During their admission, daily calcium was tested, and the patients received daily doses of calcium supplements. We test their thyroid hormone levels, and the results came back normal. Electromyography (EMG) and nerve conduction velocity (NCV) were performed to assess their nervous system. Despite experiencing paresthesia, the EMG‐NCV of all five family members was normal, and no sign of peripheral neuropathy was detected.

After completing the chelation therapy, their well‐being improved, but some symptoms of finger numbness, generalized weakness, and muscle pain remained. We follow them with checking blood lead levels (Table 4) and other electrolytes such as calcium and zinc two weeks after discharge.

**TABLE 4:** Blood lead level

	Father	Mother	Oldest child	Middle child	Youngest child
BLL (μg/dL)					
Before	51	63	41.8	37.6	35.9
After therapy	34.6	46	38.2	33.4	32.8

Abbreviation: BLL, Blood lead level.

## 3 DISCUSSION

In the presented case series, five people with the same exposure experienced different signs and symptoms. Their blood lead levels were different. They even have different outcomes after receiving the treatment. The parents’ response to treatment was significantly more favorable than children's response. One theory is that because of higher bone density in children, they absorb more lead. Children have higher lead body burden, and after chelation therapy, the excessive lead releases in the bloodstream. This can explain the children's higher lead level, compared to the parents, after chelation therapy. The younger daughter and son were experiencing attention‐deficit and difficulty in concentration. One of the reasons for this finding could be that these children were students, and their families and teachers monitored their school function. So, any changes in their cognitive and intellectual function would be noticed.

Lead poisoning is one of the medical diagnoses that is usually dismissed. Many cases with LP were investigated for other common causes of abdominal pain, anemia, and neurologic symptoms. Many unnecessary medical procedures such as endoscopy, colonoscopy, bone marrow aspiration, and neurologic imaging were performed. In such typical cases, checking for blood lead levels can become helpful. Considering the recent worldwide pandemic, they attributed their signs and symptoms to mild coronavirus (COVID‐19) infection. This situation postponed the diagnosis. The oldest child who bought the swamp stone powder suspected that they were using this powder by mistake in their food and explained this to their primary physician. Their blood lead level was tested, and the results suggested LP. They were referred to our LP clinic for further evaluation and treatment.

These findings showed that every patient with unspecific complaints and suspicious elad exposure needs to be evaluated for LP. As clinicians, we should consider heavy metal poisoning as a reasonable differential diagnosis. It is essential to find the source of LP and report it to the responsible organization. These actions can prevent many people from having exposure to lead sources. The known sources of lead are usually soil and water, old pipes, and leaded dishware, but we should consider new ways of LP, such as swamp stone in the presented study, can happen. With five‐month exposure, the blood lead level became toxic, and the patients became symptomatic. The high level of lead oxide in this product makes it a potential source for LP.

We suggest that the purchase of swamp stone powder become restricted. Only the drugstores and specific stores should be allowed to sell the product. All products should also have labels that explain swamp stone ingredients and the dangers of inappropriate consumption. This product should be kept out of reach of children. The product should have a "not eatable" sign on the label. The packaging and coloring of the product should be distinguishable from spices and other eatable materials. These cautions can help prevent accidental swamp stone poisoning.

## 4 CONCLUSION

It is vital to find sources for LP and educate the population about this heavy metal danger. Also, the government should be aware of this critical matter and evaluate food and water supplies. Population‐based studies about normal lead levels and LP are neglected in our country, Iran. The epidemiologic studies for evaluating blood lead levels in the Iranian population can help develop a guideline for screening in danger populations and providing treatment. The importance of this matter comes to light when considering the younger generation. The effect of high lead levels in children and adolescents’ cognitive and behavioral development is usually irreversible, so early diagnosis is essential. The best way to achieve this goal is a proper screening schedule.

## CONFLICT OF INTEREST

None.

## AUTHOR CONTRIBUTIONS

Dr. Nazanin Zamani and Dr. Abbas Aghabikloo gather the information and prepare the manuscript.

## ETHICAL APPROVAL

The institutional review board approval is not required for case reports. Although due to ethical principles and to keep patients’ rights protected, the names were not mentioned in the paper.

## CONSENT

The written consent was filled and signed by parents for publication of data.

## Data Availability

The data that support the findings of this study are available on request from the corresponding author. The data are not publicly available due to the fact that their containing information could compromise the privacy of research participants.
